# Editorial Note: Functional Cell Types in Taste Buds Have Distinct Longevities

**DOI:** 10.1371/journal.pone.0314739

**Published:** 2024-11-26

**Authors:** The PLOS ONE Editors

This notice is issued to share information provided by the authors regarding the preparation of Figures 1 and 7 of [1].

Regarding Figure 1:

The images in this figure show representative examples from clones imaged at different confocal settings. Aside from cropping to isolate the region of interest, the images shown were not adjusted (e.g. color, brightness, contrast) in preparing the figures for publication.

The authors note that the panels in this figure are not meant to be compared to one another in a quantitative manner, but rather serve as qualitative data showing clones of different sizes so as to illustrate and validate the EdU method that was used in subsequent experiments.

Regarding Figure 7:

Panels A-C and panels D-F present two examples of paired cells that are the product of recent mitosis. The authors state that because the cells in panels D-F separated at an angle relative to the imaging plane, their two nuclei are centered in different planes across the Z-dimension. To visualize the two separated neighboring nuclei in a single plane selected for the figure, the background of Figure 7F was reduced evenly across the entire image.
